# Delayed-Onset Hemothorax Following Cough-Induced Rib Fracture

**DOI:** 10.1155/crpu/7977884

**Published:** 2025-04-03

**Authors:** Katrina Villegas MD, Arielle Aiken MD, Mohammed Halabiya MD, Mourad Ismail MD

**Affiliations:** ^1^Department of Internal Medicine, St. Joseph's University Medical Center, Paterson, New Jersey, USA; ^2^Department of Internal Medicine, St. Joseph's Wayne Medical Center, Wayne, New Jersey, USA; ^3^Department of Pulmonary and Critical Care Medicine, St. Joseph's University Medical Center, Paterson, New Jersey, USA

## Abstract

Hemothorax, the accumulation of blood in the pleural space, is most frequently linked to chest trauma but can occasionally result from nontraumatic causes such as persistent or forceful coughing. Cough-induced rib fractures are rare, with an even less frequent association with hemothorax. We describe a case involving a 57-year-old male who presented with a worsening cough and left-sided pleuritic chest pain despite prior antibiotic and symptomatic treatment. Initial imaging revealed a minimally displaced 10th rib fracture, left-sided atelectasis, and trace pleural effusion. During his hospital stay, he developed acute respiratory distress and increased chest pain, with repeat imaging revealing a large left-sided hemothorax. Chest tube placement drained 1100 mL of blood, supporting the diagnosis of hemothorax, as evidenced by clinical presentation and imaging, despite the absence of fluid analysis. The patient's condition improved following the intervention, and he was discharged in stable condition without recurrence of hemothorax on follow-up imaging. This case highlights the rare association between cough-induced traumatic rib fractures and delayed development of hemothorax. While rib fractures typically result from blunt trauma, they can also occur from stress or repetitive coughing. Complications such as hemothorax are uncommon but potentially life-threatening. The interval development of hemothorax, as seen in this patient, underscores the importance of serial monitoring in cases of rib fractures with ongoing symptoms. Clinicians should maintain a high index of suspicion for hemothorax in patients presenting with rib fractures and persistent cough, particularly in the context of worsening respiratory symptoms or pleuritic chest pain. Early recognition and timely intervention are critical to optimizing outcomes and mitigating the risks of rapid clinical deterioration.

## 1. Introduction

A hemothorax is defined as the accumulation of blood within the pleural space, most commonly resulting from blunt or penetrating chest trauma. However, it can also occur secondary to nontraumatic causes, such as forceful or persistent coughing, which is a much rarer etiology [1]. This report presents a 57-year-old male who developed a left-sided hemothorax requiring chest tube placement, following a cough-induced rib fracture. The interval development of hemothorax in this patient's case provides an opportunity to explore atypical presentations and reinforce the importance of vigilant monitoring.

While traumatic hemothorax is well-documented, the occurrence of hemothorax secondary to rib fractures in the absence of significant trauma—particularly in the context of pneumonia—remains a gap in the existing literature. This case underscores the typical causes of hemothorax, emphasizing its strong association with trauma, including minimally displaced rib fractures. The unique circumstances of this case, including the delayed presentation of hemothorax, highlight a gap in the current understanding of cough-induced rib fractures and their complications. Further exploration of these atypical presentations is warranted to guide early recognition and management.

## 2. Case Presentation

A 57-year-old obese male with a history of hypertension presented to the emergency department (ED) with a 2-week history of productive cough. He reported intermittent bouts of coughing, producing small amounts of yellowish phlegm, accompanied by subjective fevers and chills. Initially, he sought care at an urgent care center, where he was prescribed azithromycin, prednisone, benzonatate, and guaifenesin. Despite this treatment, his symptoms persisted, prompting a return 3 days later, at which point he was prescribed an albuterol inhaler. Over the next few days, the patient developed severe, left-sided chest pain, described as sharp and pleuritic, exacerbated by coughing and movement. He revisited the ED, where he was given cyclobenzaprine and advised to take acetaminophen and apply ice packs.

**Figure 1:**
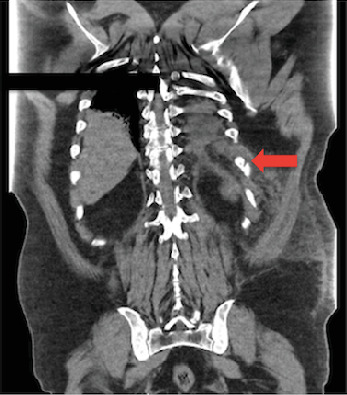
Computed tomography (CT) of the chest coronal view on admission shows minimally displaced transverse fracture of the 10^th^ rib (red arrow).

He represented to the ED 2 days later for worsening chest pain. On evaluation, the patient was in mild respiratory distress. He was mildly hypertensive, tachycardic, and tachypneic (respiratory rate 22 breaths/min) with an oxygen saturation of 95% on room air. Physical examination revealed left lower chest wall ecchymosis and tenderness, with bilateral coarse crackles most pronounced in the left lower lung field. Laboratory results were notable for leukocytosis with a left shift (18,000/*μ*L), mild hyponatremia (134 mmol/L), hyperchloremia (117 mmol/L), and an elevated erythrocyte sedimentation rate (30 mm/h). His SARS-CoV-2 test was negative. A chest X-ray revealed left lower lobe nodular opacities without effusion. A computed tomography scan of the chest (Figure 1) showed a minimally displaced left 10th rib fracture, trace left-sided pleural effusion and atelectasis. Septic workup, including blood cultures, sputum cultures, procalcitonin, and a pneumonia panel, was initiated. The patient was empirically started on ceftriaxone for presumed postviral secondary bacterial pneumonia. Morphine was administered for pain control, and the patient was admitted for further evaluation and management. Cultures remained negative, but the pneumonia panel was positive for adenovirus and rhinovirus. Due to suspected coexisting asthmatic bronchitis, he was treated with methylprednisolone, ipratropium–albuterol, and budesonide nebulization. Incentive spirometry and analgesics were continued for the rib fracture.

**Figure 2:**
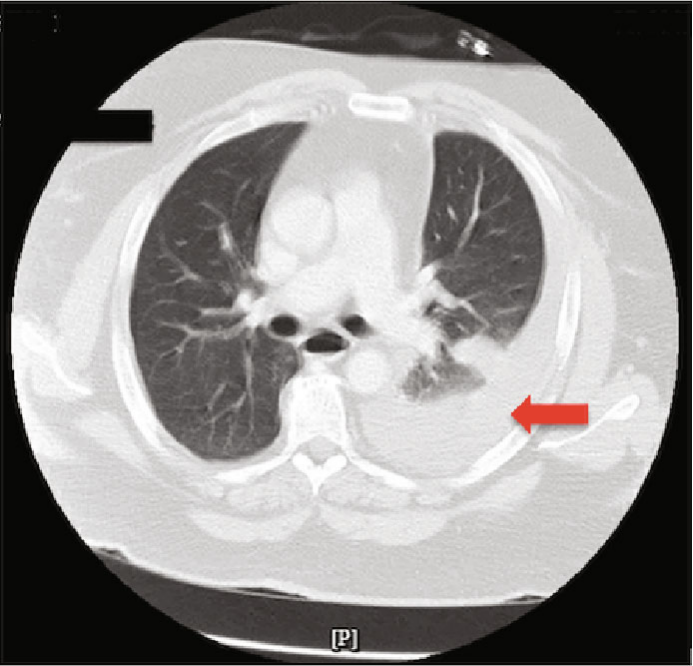
CT of the chest axial view done on Day 5 shows a large left pleural effusion (red arrow) with no contusion observed.

On hospital Day 5, the patient experienced worsening respiratory distress and increased left-sided chest pain. Repeat imaging revealed a new large left-sided hemothorax (Figure 2). The cardiothoracic surgery (CTS) team was consulted, and a chest tube was placed, draining 1100 mL of dark red blood. The patient's serum hemoglobin prior to the chest tube placement was at 12.9 g/dL, which dropped to 10.1 g/dL a day postprocedure. Although pleural fluid analysis was not performed, the large volume of blood drained, combined with the observed drop in serum hemoglobin by 2.8 g/dL and clinical course, strongly supports the diagnosis of traumatic hemothorax. Following chest tube placement, the patient experienced gradual relief of symptoms, with a marked decrease in chest pain and respiratory distress over the next few days.

The patient's status and chest pain improved over the subsequent days, with decreased chest tube output. Follow-up imaging showed resolution of the hemothorax, allowing for chest tube removal. The patient was discharged on analgesics with close outpatient follow-up with the CTS team. At discharge, his respiratory symptoms were significantly improved, and imaging showed no recurrence of hemothorax.

## 3. Discussion

Hemothorax, defined as the accumulation of blood in the pleural space, is mostly associated with blunt or penetrating chest trauma. However, nontraumatic etiologies, while rare, include malignancies, pulmonary arteriovenous fistulas, coagulopathies, and vascular abnormalities such as ruptured intercostal or internal mammary arteries. The blood source may originate from extra pleural structures, such as intercostal arteries, or intrapleural structures, including the aorta, pulmonary veins, or azygos vein [2, 3]. Patients typically present with pleuritic chest pain, respiratory distress, and signs of hemodynamic instability, emphasizing the critical need for early recognition and intervention.

Rib fractures are most frequently caused by blunt chest trauma but can also result from pathological fractures secondary to malignancy, metabolic bone disease, or stress fractures induced by forceful or prolonged coughing [4, 5]. These fractures can lead to complications including pneumothorax, flail chest, pulmonary contusion, and, less commonly, hemothorax. Cough-induced rib fractures are primarily identified in postmenopausal women with reduced bone density, suggesting a link to osteoporosis or osteopenia [6, 7]. In contrast, this case involved a male patient, underscoring the importance of recognizing that this condition can occur across diverse patient populations.

Our patient's case is particularly notable for the interval development of a hemothorax 5 days after the diagnosis of a rib fracture on admission. Although rib fracture-induced hemothorax has been documented, the delayed onset observed here is highly unusual. As seen in prior reports, including the case described by Camarillo-Reyes et al. [1], hemothorax typically develops immediately resulting from intercostal vessel disruption following rib fractures caused by severe coughing. However, our case highlights an unusual delayed onset, which may suggest a progressive vascular injury or inflammatory and infectious processes [2]. The absence of pleural fluid analysis, in this case, is a limitation, but the large volume of blood drained and the clinical course strongly supported the diagnosis of traumatic hemothorax. Additionally, the observed 2.8 g/dL drop in serum hemoglobin from the day prior to chest tube placement to 1 day postprocedure further corroborates the diagnosis. This significant hemoglobin drop is consistent with ongoing blood loss, reinforcing the clinical suspicion of hemothorax, even in the absence of pleural fluid analysis.

Coughing, while often dismissed as a benign symptom of respiratory infections, can have serious sequelae, including rib fractures [4, 5] and hemothorax [2, 3, 8]. The interval development of hemothorax in this case underscores the importance of serial monitoring and imaging in patients presenting with rib fractures and persistent cough. Early recognition and timely intervention—such as chest tube placement and hemodynamic stabilization—are essential to preventing life-threatening complications.

A severe cough should not be underestimated, particularly when it leads to structural complications such as rib fractures. Hemothorax, with its potential for rapid hemodynamic instability, requires a high index of suspicion in patients presenting with worsening respiratory symptoms or pleuritic chest pain. This case highlights the rare association between cough-induced traumatic rib fractures and the development of hemothorax. The delayed onset of hemothorax in this case highlights the potential for complications in patients with rib fractures, especially those with ongoing respiratory symptoms. This delayed progression could lead to missed diagnoses or delayed interventions, underscoring the importance of close monitoring and timely follow-up in such cases.

## Data Availability

The data that support the findings of this study are available from the corresponding author upon reasonable request.
